# Psychological Morbidity After Ocular Trauma: Association Between Initial Visual Loss and PTSD

**DOI:** 10.3390/diagnostics16040639

**Published:** 2026-02-23

**Authors:** Gamze Ucan Gunduz, Oguzhan Kilincel, Sema Nizam Tekcan, Cengiz Akkaya, Ozgur Yalcinbayir

**Affiliations:** 1 Department of Ophthalmology, School of Medicine, Bursa Uludag University, Bursa 16059, Turkey; nizamsema@gmail.com (S.N.T.); yalcinbayir@yahoo.com (O.Y.); 2 Faculty of Health Sciences, Istanbul Gelişim University, Istanbul 34310, Turkey; okilincel@gmail.com; 3 Department of Psychiatry, School of Medicine, Bursa Uludag University, Bursa 16059, Turkey; cakkaya@uludag.edu.tr

**Keywords:** anxiety, depression, mechanical ocular trauma, initial visual acuity, post-traumatic stress disorder

## Abstract

**Background:** Ocular trauma is a significant cause of monocular visual impairment and potential psychological morbidity. This study aimed to determine the prevalence of anxiety, depression, and post-traumatic stress disorder (PTSD) in patients with mechanical ocular trauma and to investigate the predictive value of baseline clinical characteristics, specifically initial visual acuity. **Methods:** This retrospective study included 58 adult patients treated for mechanical ocular trauma. Sociodemographic data, injury mechanisms, and clinical variables, including initial visual acuity (LogMAR), ocular trauma score, and number of ocular surgeries, were analyzed. Psychological status was assessed using the Beck Depression Inventory, Beck Anxiety Inventory, and a PTSD checklist. Multivariate logistic regression and correlation analyses were performed to identify predictors of severe PTSD. **Results:** The cohort was predominantly male (86.2%) with a mean age of 42.5 years. Severe or very severe PTSD symptoms were identified in 35.1% of patients. Analysis revealed a significant positive correlation between initial visual acuity and PTSD scores (r = 0.273, *p* = 0.038). In the logistic regression model, initial visual acuity (LogMAR) demonstrated the highest odds ratio for severe PTSD in the multivariable model; however, this association did not reach statistical significance (OR = 2.164, 95% CI: 0.720–6.508, *p* = 0.169) and should therefore be interpreted as an exploratory trend rather than a confirmed predictor. **Conclusions:** Greater visual loss at the time of injury showed the strongest, although non-significant, association with subsequent PTSD symptom severity. These findings suggest that patients with severe initial visual impairment following ocular trauma may benefit from early psychological screening and timely mental health referral, warranting confirmation in larger prospective studies.

## 1. Introduction

Ocular trauma is a leading cause of unilateral vision loss, with over 55 million eye injuries reported globally each year. Among these cases, approximately 19 million result in at least partial permanent vision loss in one eye, while 1.6 million people suffer complete blindness due to their injuries [1]. In particular, mechanical ocular injuries constitute a major contributor to monocular visual impairment and blindness worldwide, resulting in profound consequences that extend beyond physical disability to include significant functional, esthetic, and psychosocial deterioration [2].

The sudden loss of visual function and the potential for facial disfigurement can precipitate a wide range of psychological comorbidities, including social avoidance, anxiety, and depression [3]. While the physical management of these injuries is well-established through protocols such as the ocular trauma score (OTS), the psychiatric sequelae, particularly post-traumatic stress disorder (PTSD), often remain underdiagnosed and undertreated in ophthalmological practice [4].

The prevalence of PTSD in the general population following major trauma is estimated to be between 20% and 30%, yet individuals with visual impairments face unique vulnerabilities [5]. Current literature suggests that the prevalence of PTSD among visually impaired individuals ranges broadly from 4% to 50%, depending on the nature of the trauma and the population studied [6]. A critical mechanism proposed in this context is “Information Deprivation Trauma,” whereby the abrupt loss of visual sensory input during a traumatic event disrupts thalamocortical processing and limbic system activity. This sudden sensory deprivation may impair adaptive cortical prediction mechanisms, exacerbating feelings of helplessness, unpredictability, and loss of control, and thereby increasing vulnerability to trauma-related psychological symptoms such as re-experiencing and hyperarousal [6,7]. Furthermore, ocular trauma is frequently associated with intentional injuries or workplace accidents, factors that have been independently linked to higher PTSD symptom severity compared to accidental injuries [6,8].

Despite the established link between general trauma and PTSD, the relationship between specific clinical ocular parameters, such as initial visual acuity (VA) or the number of surgeries, and psychiatric outcomes remains complex and occasionally contradictory. For instance, Li et al. [9] recently reported that patients with mild visual impairment might exhibit higher anxiety levels compared to those with blindness, attributing this to the uncertainty of prognosis and persistent pain rather than the vision loss itself. Conversely, in pediatric populations, increased surgical interventions have been correlated with lower quality of life and psychosocial functioning [10]. However, the predictive value of standard clinical metrics like the OTS or initial visual acuity specifically for the development of severe PTSD in adult mechanical trauma patients has not been sufficiently elucidated.

While the OTS has proven to be a robust tool for predicting final visual acuity, its utility in forecasting psychiatric morbidity is less understood. Previous studies have focused largely on demographic risk factors, such as female gender and history of psychiatric illness, or injury mechanisms like penetrating versus blunt trauma [11]. There is a paucity of data examining whether the magnitude of initial visual deprivation acts as a “traumatic anchor”, potentially predicting the severity of post-traumatic stress independent of the final visual outcome or the number of reconstructive surgeries performed.

The aim of this study was to investigate the association between the severity of visual loss at the time of injury and subsequent post-traumatic stress disorder (PTSD) symptom severity in patients hospitalized for ocular trauma. Specifically, the objectives of the study were:

- To evaluate the relationship between initial visual acuity at the time of injury and subsequent PTSD symptom severity.
- To examine the association of PTSD symptom severity with final visual outcomes and surgical burden.
- To identify early clinical predictors that may help guide timely psychological screening and intervention in high-risk patients.

Understanding these predictors may facilitate the integration of early psychological assessment into routine ophthalmologic trauma care.

## 2. Material and Methods

### 2.1. Study Design and Participants

**Figure 1:**
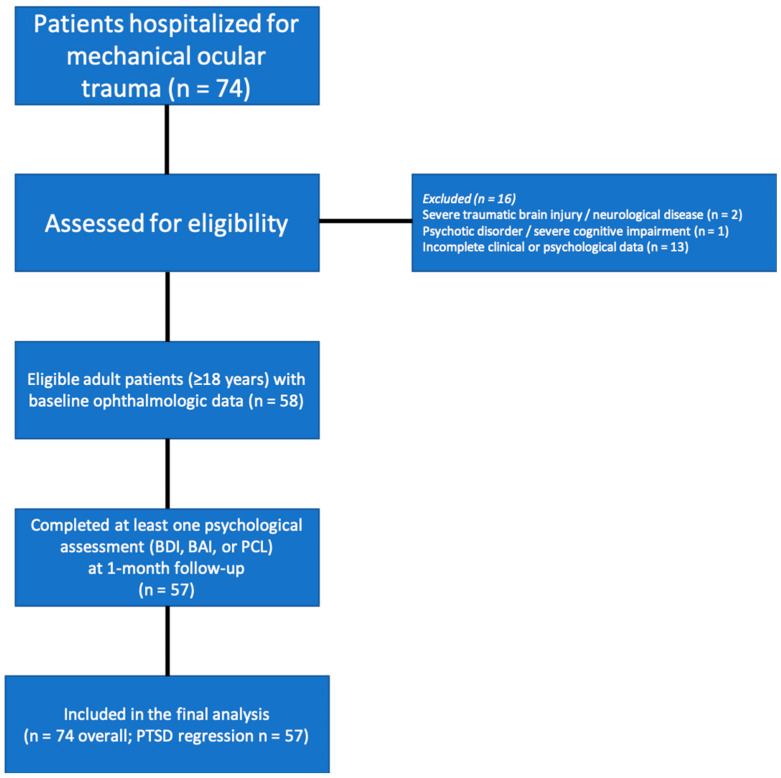
The study flowchart.

Adult patients (≥18 years) who were hospitalized for mechanical ocular trauma and had available baseline ophthalmologic data, including initial visual acuity, were eligible for inclusion. Patients were required to have sufficient clinical information and to have completed at least one psychological assessment during follow-up. Exclusion criteria included the presence of severe traumatic brain injury or neurological disease, documented psychotic disorders or severe cognitive impairment that could interfere with self-report assessments, and incomplete clinical or psychological data (Figure 1). Sociodemographic data were recorded for all participants, including age, gender, marital status, education level, and employment status. Detailed injury histories were obtained to classify the trauma mechanism (e.g., occupational accident, sports injury, domestic accident, assault) and the nature of the injury (penetrating, perforating, blunt, or chemical). Patients with missing data were excluded from the analysis (complete-case approach).

Ophthalmologic evaluations, including visual acuity, biomicroscopy, and fundus examination, were performed at baseline and at 1 and 6 months following ocular trauma, whereas psychological symptom assessments were conducted at 1 month after the trauma.

Our study was approved by the Uludağ University Faculty of Medicine Clinical Research Ethics Committee (No: 2020-1/23, date: 20 January 2020).

### 2.2. Clinical Ophthalmologic Assessment

All patients underwent a comprehensive ophthalmologic examination. The following clinical parameters were recorded and analyzed:

**Visual Acuity (VA):** Visual acuity was measured at the time of initial presentation (initial VA), at 1 month, and at 6 months post-injury. Visual acuity measurements recorded in Snellen format were converted to logarithm of the minimum angle of resolution (LogMAR) units for statistical analysis, as LogMAR provides a linear and standardized scale in which higher values indicate poorer visual acuity [12,13].

**Ocular Trauma Score (OTS):** The OTS was calculated for each patient to predict visual outcomes using a standardized system derived from the Birmingham Eye Trauma Terminology (BETT) [14].

**Surgical History:** The total number of ocular surgeries performed per patient was recorded to evaluate the burden of surgical intervention.

### 2.3. Psychological Assessment Tools

Psychological morbidity was assessed using self-report questionnaires focusing on depression, anxiety, and post-traumatic stress symptoms.

**Depression:** The Beck Depression Inventory (BDI) was used to evaluate the severity of depressive symptoms. The BDI is a 21-item self-report questionnaire with total scores ranging from 0 to 63. Scores were categorized as minimal (0–13), mild (14–19), moderate (20–28), and severe depression (29–63) [15,16].

**Anxiety:** The Beck Anxiety Inventory (BAI) was administered to measure anxiety levels, with scores similarly categorized from minimal to severe. The BAI is a 21-item self-report questionnaire with total scores ranging from 0 to 63. Scores were categorized as minimal (0–7), mild (8–15), moderate (16–25), and severe anxiety (26–63) [17,18].

**Post-Traumatic Stress Disorder (PTSD):** PTSD symptoms were assessed using the Turkish validated version of the PTSD Checklist (PCL), a widely used self-report instrument for evaluating post-traumatic stress symptoms. The scale consists of items rated on a Likert-type scale, yielding a total score ranging from 0 to 80, with higher scores indicating greater symptom severity. In accordance with previous validation studies, PTSD severity was categorized as none/minimal, mild-to-moderate, severe, and very severe. For the primary outcome analysis, a cut-off score of ≥40 was used to define severe or very severe PTSD symptoms [19,20].

### 2.4. Statistical Analysis

All statistical analyses were performed using the Python (Python Software Foundation, Wilmington, DE, USA) programming environment. Continuous variables were converted to appropriate numerical formats, and analyses were conducted using a complete-case approach, excluding observations with missing data. Chi-square analysis was used for sociodemographic and clinical characteristics distributions. The primary outcome of this study was defined as a binary variable based on the severity of post-traumatic stress symptoms. For the primary analysis, “severe” and “very severe” groups were combined, and a PTSD score\geq 40 was defined as the “event”. A multivariate logistic regression model was established to predict the presence of severe/very severe PTSD. The model included variables considered clinically significant: ocular trauma severity (OTS score), the number of ocular surgeries, age, sex, binary indicators representing trauma subtypes, and initial VA (LogMAR). To address class imbalance in the outcome variable, the model was fitted using balanced class weights. Model coefficients were estimated on the logit scale, and the results were reported as Odds Ratios (OR) with 95% confidence intervals (CI), calculated using the Wald test via the estimated variance-covariance matrix. The discriminative ability of the model was evaluated using the Receiver Operating Characteristic (ROC) curve, and the Area Under the Curve (AUC) was reported as a summary metric. Rather than imposing a fixed classification threshold (0.50), the optimal probability threshold was determined by maximizing the Youden’s J index (sensitivity + specificity − 1) to identify a decision point more suitable for clinical application. At this optimal threshold, sensitivity, specificity, positive/negative predictive values (PPV/NPV), accuracy, and a confusion matrix (TN, FP, FN, TP) were reported. For all analyses, a two-tailed *p* < 0.05 was considered statistically significant. Results are presented in a standard reportable format, including performance summaries, confusion matrices, and an OR table.

## 3. Results

The baseline sociodemographic and clinical characteristics of the study population are presented in Table 1. The mean age of participants was 42.5 ± 14.3 years (n = 58), indicating a predominantly working-age cohort. The sample was overwhelmingly male (86.2%), with females comprising 13.8%. Most participants were married (69.0%), while 24.1% were single and 6.9% divorced. Regarding employment, 41.4% were workers and 36.2% were unemployed. Educational attainment was generally low to moderate, with the largest groups reporting high school (31.0%) and primary school (25.9%) education. A minority reported a psychiatric history (10.3%), and prior accidents were reported by 13.8% of the cohort. Detailed category distributions, along with χ^2^ statistics and *p*-values, are provided in the table.

Although the study cohort consisted of patients hospitalized for ocular trauma at a tertiary referral center, the demographic and injury characteristics were broadly consistent with those reported in previous epidemiological studies of ocular trauma. Nevertheless, as the sample represents a hospital-based population with potentially more severe injuries, the findings may not fully reflect the entire spectrum of ocular trauma seen in the general population [1,21].

The distribution of the mechanisms of injury among the patients is summarized in Table 2. The most frequent causes of ocular trauma were occupational accidents and domestic accidents, each accounting for 34.5% (n = 20) of the cases. Together, these two categories constituted 69% of the total trauma etiology. Accidental injuries caused by another person followed as the third most common mechanism, representing 17.2% (n = 10) of the population. Other mechanisms were observed at lower frequencies: assault accounted for 5.2% (n = 3), while animal-inflicted injuries and sports injuries each represented 3.4% (n = 2) of the cases. The least frequent mechanism recorded was firearm injuries, which accounted for 1.7% (n = 1) of the total cases.

Table 3 presents the distribution of ocular trauma cases based on laterality, type of injury, and the specific tissues damaged. The data reveal a nearly equal distribution between the right and left eyes, with the left eye being slightly more frequently affected (51.7%). Regarding the mechanism of injury, open-globe injuries, specifically penetrating traumas, were the most prevalent, accounting for 65.5% of the total cases.

The mean OTS was 68.03 ± 16.35, with values ranging from 26.00 to 100.00. Visual acuity at presentation was 2.56 ± 0.97 LogMAR (range: 0.10–3.10) and 51 patients (88.0%) had a visual acuity of less than 0.1, corresponding to severe visual impairment. At the one-month follow-up, the mean visual acuity was 1.72 ± 1.12 LogMAR (range: 0.05–3.10), and at the six-month follow-up, it was 1.23 ± 1.18 LogMAR (range: 0.00–3.10). The mean number of surgical interventions per patient was 2.62 ± 1.64, ranging from 0 to 7. The descriptive statistics for the OTS and visual acuity-related continuous variables are summarized in Table 4.

The mean score for the BDI was 9.8 ± 9.9, with values ranging from 0.0 to 33.0. The mean PTSD score was 29.4 ± 24.0, with scores ranging between 0.0 and 87.0. For the BAI, the mean score was 10.2 ± 11.9 (range: 0.0–41.0, n = 58). When categorized according to established cut-off points, the majority of the patients exhibited minimal depressive symptoms; specifically, 56.9% were classified as minimal, 17.2% as mild, 20.7% as moderate, and 5.2% as severe depression. Similarly, BAI scores indicated that a substantial proportion of the participants had minimal anxiety levels (58.6%), while 10.3% were categorized as mild, 20.7% as moderate, and 10.3% as severe anxiety. Regarding the severity of PTSD symptoms, 42.1% of the patients were classified as none/minimal, 22.8% as mild-to-moderate, 21.1% as severe, and 14.0% as very severe. The categorical distribution of BDI, BAI, and PTSD levels is summarized in Table 5.

Table 6 presents the correlations between BDI, PTSD, and BAI scores with clinical parameters, including OTS, visual acuity at presentation, the 1st and 6th months, and the number of surgical interventions. According to the results, no statistically significant correlation was observed between OTS and psychological scale scores (BDI: r = −0.120, *p* = 0.369; PTSD: r = −0.199, *p* = 0.135; BAI: r = −0.163, *p* = 0.222). Similarly, no significant correlations were found between 1-month and 6-month visual acuity and BDI, PTSD, or BAI scores (all *p* > 0.05). In contrast, a weak but statistically significant positive correlation was observed between visual acuity at presentation and PTSD scores (r = 0.273, *p* = 0.038). This finding suggests that as the PTSD score increases, the LogMAR value at presentation also tends to increase (indicating poorer initial visual acuity). No significant relationship was identified between initial visual acuity and BDI (r = 0.166, *p* = 0.213) or BAI scores (r = 0.133, *p* = 0.319).

In this study, a binary outcome variable was created to predict the presence of severe/very severe PTSD in ocular trauma cases, with the dependent variable defined as a PTSD score > 40. Logistic regression was employed for the modeling process. The analysis was conducted on 58 cases, of which 20 (34.5%) were in the “severe/very severe” (PTSD > 40) group (Table 7). Multicollinearity among independent variables was assessed using Variance Inflation Factors (VIF). All variables showed VIF values < 4.0 (highest VIF = 3.62 for OTS), indicating no significant multicollinearity that would compromise the stability of the logistic regression model. The model’s discriminative performance in this sample was moderate, with an ROC-AUC of 0.761. The optimal probability threshold determined via the Youden’s J index was 0.48. At this threshold, the model demonstrated a sensitivity of 0.80 and a specificity of 0.68. Additionally, the positive predictive value (PPV) was 0.571, the negative predictive value (NPV) was 0.867, and the overall accuracy was 0.724. These results indicate that the model performs more robustly in excluding negative cases (high NPV), while providing moderate precision in positive classification (PPV). Given the limited number of severe PTSD events relative to the number of covariates, these performance estimates should be interpreted as exploratory.

None of the trauma mechanism categories demonstrated a statistically significant association with severe PTSD outcomes (PTSD score > 40) (all *p* > 0.05). These findings suggest that, within our cohort, the mechanism of injury alone was not an independent predictor of severe PTSD symptoms after adjustment for clinical and demographic variables.

## 4. Discussion

Our study revealed that 35.1% (n = 20) of the 58 patients suffering from mechanical ocular trauma exhibited severe or very severe symptoms of PTSD, with 86.2% of the cohort being male. In our analyses, while no statistically significant relationship was found between final visual acuity or the number of surgeries performed and psychological morbidity, a significant positive correlation was detected between initial visual acuity (presenting LogMAR) and PTSD scores (r = 0.273, *p* = 0.038). In the logistic regression model, initial visual acuity showed the highest odds ratio among the examined variables; however, this effect did not reach statistical significance and should be interpreted as an exploratory signal rather than a statistically confirmed predictor.

The 35.1% rate of severe PTSD identified in our study aligns with the systematic review by Ham et al., which reports PTSD prevalence in visually impaired individuals ranging widely from 4% to 50% [6]. Furthermore, when compared to data from Haagsma et al., who reported a PTSD rate of roughly 20–30% in the general major trauma population one year post-injury, our findings indicate that ocular trauma patients fall at the upper end of this spectrum [22]. Our sociodemographic data confirm that ocular trauma is concentrated particularly among young-to-middle-aged males (mean age 42.5), resulting primarily from occupational and sports accidents (86.2% male, 34.5% occupational accidents). This profile parallels epidemiological data from Baker et al., who noted that workplace injuries have the highest incidence among men in this age demographic [23]. However, this gender imbalance may limit the generalizability of the findings to female patients and should be considered when interpreting the results, as potential sex-related differences in psychological responses to trauma cannot be excluded.

The absence of a significant association between surgical burden and PTSD symptom severity may partly reflect age-related differences in psychological resilience. Younger individuals may exhibit greater adaptive coping capacity, neuroplasticity, and social support engagement, which could mitigate the psychological impact of repeated surgical interventions. Conversely, resilience patterns in older patients may be shaped by comorbidities, life experience, and established coping strategies. These age-related resilience factors may therefore moderate the psychological consequences of surgical burden and warrant further investigation in larger longitudinal studies.

One of the key observations of this study was that poorer visual acuity at the time of trauma was associated with greater PTSD symptom severity; however, this association did not remain statistically significant after adjustment for clinical variables and should therefore be interpreted cautiously. A potential explanatory framework is the “Information Deprivation Trauma” hypothesis proposed by Ham et al., which suggests that the abrupt loss of visual sensory input during the traumatic event may intensify feelings of uncertainty, helplessness, and loss of control, thereby deepening the traumatic impact of the event [6]. From a neurobiological perspective, sudden sensory deprivation may disrupt thalamocortical and limbic system processing and impair adaptive predictive mechanisms, amplifying early stress responses. This framework may help explain why the severity of visual loss at the time of injury could exert a greater psychological influence than the eventual long-term visual outcome, which may be partially mitigated through subsequent psychological and functional adaptation. Although Li et al. suggest that anxiety may be higher in patients with mild visual impairment due to uncertainty and persistent pain [9], our results suggest that regarding PTSD specifically, the severity of the initial trauma (the immediate sense of blindness) may reflect the subjective severity of the traumatic experience at the time of injury. Additionally, considering Barker-Collo et al.’s finding that increased duration of loss of consciousness predicts PTSD severity in traumatic brain injury, the sensory deprivation in ocular trauma may act as a similar marker of trauma severity [24].

From a temporal perspective, the abrupt “immediate sense of blindness” at the time of injury may primarily trigger an acute stress response characterized by heightened autonomic arousal, fear, and loss of control. However, when this sensory deprivation is perceived as overwhelming and remains cognitively unprocessed, it may contribute to the consolidation of traumatic memory traces, thereby increasing the risk of persistent or delayed PTSD symptoms. In contrast to other forms of trauma where sensory input remains intact, the sudden disruption of visual feedback may intensify early stress reactivity while simultaneously impairing adaptive cognitive integration of the event. This dual mechanism may partially explain why the magnitude of initial visual loss appears clinically relevant in the early psychological trajectory following ocular trauma.

In line with broader environmental stress models, Liu et al. [25] recently reported that environmental contexts are associated with distinct neural and psychopathological signatures, highlighting how external environments—whether social or physical—shape brain connectivity patterns and emotional regulation profiles [25]. These findings suggest that traumatic ocular injury should also be conceptualized within a broader environmental stress framework, in which sudden sensory deprivation represents an extreme alteration of the individual’s perceptual environment. Thus, the psychological sequelae observed in our cohort may reflect not only the physical insult itself but also the abrupt transformation of the patient’s environmental and sensory experience.

Contradictory data exist in the literature regarding the psychological effects of the number of surgeries. Karaman et al. reported that an increased number of surgeries in pediatric patients negatively affected quality of life and psychological status [8]. However, in our study of adult patients who underwent an average of 2.62 surgeries, no significant relationship was detected between the number of surgeries and PTSD or depression (*p* > 0.05). This difference may be explained by adults possessing different coping mechanisms (resilience) than children. As noted by Chotprasert et al., adult patients can develop “psychological adjustment” by adapting to ocular prostheses or rehabilitation processes, accepting the surgical process as a necessary part of recovery rather than a compounding trauma [26].

On the other hand, recovery following traumatic injury is increasingly understood as a dynamic process involving adaptive neural plasticity and compensatory network reorganization after sensory or neurological insults. Evidence from broader neurological injury literature suggests that such compensatory pathways may influence both functional recovery and psychological adjustment, indicating that post-traumatic outcomes after ocular injury may reflect not only structural damage but also ongoing neuroadaptive processes [27].

Our study has certain limitations. First, the relatively small sample size (n = 58) may have limited statistical power, particularly in multivariate logistic regression analyses; indeed, although the initial VA showed a clinically significant Odds Ratio (2.164), it remained on the borderline of statistical significance (*p* = 0.169). Although the effect size was relatively large (OR = 2.164), the lack of statistical significance and wide confidence intervals indicate that this observation should be considered hypothesis-generating. The concept of initial ‘visual deprivation’ acting as a traumatic anchor therefore remains a theoretical interpretation requiring confirmation in larger prospective samples. The wide confidence intervals observed are likely a reflection of our limited sample size and the low number of severe PTSD events. As such, our findings should be interpreted as a non-significant clinical trend rather than a definitive predictive rule.

Second, the retrospective nature of the study and the reliance on self-report scales for psychological assessment may introduce response bias [9]. Furthermore, the lack of detailed information regarding patients’ pre-trauma psychiatric history limits the ability to attribute post-trauma psychological symptoms solely to the injury, although 89.7% of the cohort reported no prior psychiatric history. In addition, the absence of a control group restricts the ability to determine whether the observed psychological outcomes are specific to traumatic ocular injury or represent general responses to acute medical trauma. Future prospective studies including appropriate comparison groups, such as patients with non-traumatic acute vision loss or other forms of physical trauma, are needed to further clarify the specificity of these associations. Finally, as this study was conducted at a single center, multicenter studies with larger sample sizes are warranted to enhance the generalizability of the results.

Third, the relatively small number of severe PTSD events in relation to the number of predictors increases the risk of overfitting and limits the generalizability of the multivariable model. In addition, model performance was evaluated using the same dataset in which the model was developed; therefore, the reported AUC and optimal cut-off values may represent optimistic estimates.

In conclusion, ocular trauma patients with severe initial visual impairment may represent a subgroup with increased vulnerability to PTSD symptoms. However, this observation requires confirmation in larger, prospective studies before being translated into clinical risk stratification. Given that PTSD prevalence in this group is higher than in general trauma populations, integrating psychological screening into standard ophthalmological trauma care is essential to identify these high-risk patients early and refer them for appropriate support.

From a clinical perspective, early psychological screening may be integrated into ophthalmologic emergency department workflows by incorporating brief validated PTSD screening tools during initial hospitalization or early follow-up visits, particularly for patients presenting with severe visual loss at the time of injury. Patients identified as high-risk may then be referred for early psychiatric or psychological evaluation, enabling timely supportive interventions alongside ophthalmologic management.

## Figures and Tables

**Table 1:** Sociodemographic characteristics of the patients.

Variable	Category	Value	χ^2^ (df)	*p*
Age (years)		42.5 ± 14.3 (n = 58)	–	–
Sex	Female	8 (13.8%)	25.172 (1)	<0.001
	Male	50 (86.2%)		
Marital Status	Single	14 (24.1%)	42.690 (2)	<0.001
	Married	40 (69.0%)		
	Divorced	4 (6.9%)		
Employment Status	Worker	24 (41.4%)	18.966 (3)	<0.001
	Civil servant	2 (3.4%)		
	Retired	11 (19.0%)		
	Unemployed	21 (36.2%)		
Educational Level	Illiterate	1 (1.7%)	20.448 (5)	0.001
	Literate only	3 (5.2%)		
	Primary school	15 (25.9%)		
	Middle school	10 (17.2%)		
	High school	18 (31.0%)		
	University	11 (19.0%)		
Psychiatric History	Yes	6 (10.3%)	37.241 (1)	<0.001
	No	52 (89.7%)		
History of Prior Accidents	Yes	8 (13.8%)	25.172 (1)	<0.001
	No	50 (86.2%)		

**Table 2:** Mechanism of injury in patients with ocular trauma.

Mechanism of Injury	n	%
Occupational accident	20	34.5
Domestic accident	20	34.5
Accidental injury by another person	10	17.2
Assault	3	5.2
Animal-inflicted injury	2	3.4
Sports injury	2	3.4
Firearm	1	1.7

**Table 3:** Distribution of ocular trauma by affected eye, type of injury, and damaged tissues.

Variables	Category	n	%
Laterality	Right eye	28	48.3
	Left eye	30	51.7
Type of ocular trauma	Penetrating	38	65.5
	Perforating	1	1.7
	Blunt	10	17.2
	Chemical	3	5.2
	Intraocular foreign body	6	10.3
Damaged tissues	Cornea	34	58.6
	Sclera	34	58.6
	Lens	34	58.6
	Iris	37	63.8

**Table 4:** Descriptive statistics of continuous variables related to OTS and visual acuity.

Variable	Mean	Standard Deviation	Minimum	Maximum
Ocular Trauma Score (OTS)	68.03	16.35	26.00	100.00
Initial Visual Acuity	2.56	0.97	0.10	3.10
Visual Acuity at 1 Months	1.72	1.12	0.05	3.10
Visual Acuity at 6 Months	1.23	1.18	0.00	3.10
Number of Ocular Surgeries	2.62	1.64	0	7

Visual acuity values were given as LogMAR.

**Table 5:** Categorical distribution of Beck Depression Inventory, Beck Anxiety Inventory, and PTSD scores.

Scale	Category	n	%
Beck Depression Inventory	Minimal	33	56.9
	Mild	10	17.2
	Moderate	12	20.7
	Severe	3	5.2
Beck Anxiety Inventory	Minimal	34	58.6
	Mild	6	10.3
	Moderate	12	20.7
	Severe	6	10.3
PTSD Score	None/Minimal	24	42.1
	Mild-to-Moderate	13	22.8
	Severe	12	21.1
	Very Severe	8	14.0

**Table 6:** Correlation between clinical parameters and psychological scale scores.

Clinical Parameters	Statistics	BDI	PTSD Score	BAI
Ocular Trauma Score	rho	−0.120	−0.199	−0.163
	*p*	0.369	0.135	0.222
Initial visual acuity	rho	0.166	0.273	0.133
	*p*	0.213	0.038 *	0.319
1-Month visual acuity	rho	−0.076	0.114	0.055
	*p*	0.569	0.396	0.680
6-Month visual acuity	rho	0.014	0.095	0.118
	*p*	0.917	0.477	0.376
Number of Surgeries	rho	−0.073	0.065	−0.039
	*p*	0.585	0.627	0.772

Note: rho: Spearman’s rank correlation coefficient; *p*: *p*-value *: Correlation is statistically significant at the 0.05 level (2-tailed). BDI: Beck Depression Inventory, PTSD: Post-traumatic stress disorder, BAI: Beck anxiety inventory. Visual acuity is given as LogMAR values.

**Table 7:** Logistic regression analysis for predicting severe and very severe PTSD (PTSD Score > 40).

Model Coefficients	OR	Lower 95% CI	Upper 95% CI	*p*	VIF
Baseline visual acuity	2.164	0.720	6.508	0.169	3.51
Ocular Trauma Score	1.020	0.958	1.087	0.531	3.62
Number of Ocular Surgeries	0.937	0.626	1.087	0.752	1.51
Age	1.011	0.966	1.058	0.638	1.38
Sex (Female)	0.299	0.046	1.921	0.203	1.17
Trauma Type 1 (Sharp-Penetrating)	0.826	0.158	4.321	0.821	2.41
Trauma Type 2 (Blunt)	0.629	0.144	2.753	0.538	1.90
Trauma Type 3 (Firearm)	1.229	0.086	17.54	0.879	3.46
Trauma Type 4 (Burn/Chemical)	0.601	0.020	18.52	0.771	1.59
Intercept	0.307	0.00	288.83	0.735	-

Note: OR: Odds Ratio; CI: Confidence Interval; PTSD: Post-Traumatic Stress Disorder; LogMAR: Logarithm of the Minimum Angle of Resolution; OTS: Ocular Trauma Score; VIF: Variance Inflation Factor. The model was fitted using balanced class weights to account for class distribution. The classification threshold (0.48) was determined by maximizing the Youden J index (Sensitivity + Specificity − 1). Trauma types are coded as binary indicators (1: Yes, 0: No).

## Data Availability

The raw data supporting the conclusions of this article will be made available by the authors on request.

## Abbreviations

The following abbreviations are used in this manuscript:

VA	Visual acuity
PPV	Positive predictive value
NPV	Negative predictive value
BAI	Beck Anxiety Inventory
BDI	Beck Depression Inventory
PTSD	Post-traumatic stress disorder
LogMAR	Logarithm of the Minimum Angle of Resolution
OTS	Ocular trauma score

## References

[1] Pelletier J., Reagan K., McLeod S., Kronk N., Dickson K., Ohman K., Santos M. (2025). Epidemiology of ocular trauma in limited-resource settings: A narrative review. Front. Med..

[2] Qi Y., Zhang F.Y., Peng G.H., Zhu Y., Wan G.M., Wang W.Z., Ma J., Ren S.J. (2015). Characteristics and visual outcomes of patients hospitalized for ocular trauma in central China: 2006–2011. Int. J. Ophthalmol..

[3] Hellman J., Mahmood B., Lin L.K. (2023). Anxiety and Depression after Traumatic Open-Globe Injury. J. Emerg. Trauma. Shock..

[4] Keles A., Karayagmurlu A., Yetkin E., Sonmez K., Karatepe M.S., Karaman S.K. (2023). Development of posttraumatic stress disorder and depression after open globe injury in adults. Graefes Arch. Clin. Exp. Ophthalmol..

[5] Schincariol A., Orru G., Otgaar H., Sartori G., Scarpazza C. (2024). Posttraumatic stress disorder (PTSD) prevalence: An umbrella review. Psychol. Med..

[6] van der Ham A.J., van der Aa H.P., Brunes A., Heir T., de Vries R., van Rens G.H., van Nispen R.M. (2021). The development of posttraumatic stress disorder in individuals with visual impairment: A systematic search and review. Ophthalmic Physiol. Opt..

[7] Harricharan S., McKinnon M.C., Lanius R.A. (2021). How Processing of Sensory Information From the Internal and External Worlds Shape the Perception and Engagement With the World in the Aftermath of Trauma: Implications for PTSD. Front. Neurosci..

[8] Jiang T., Webster J.L., Robinson A., Kassam-Adams N., Richmond T.S. (2018). Emotional responses to unintentional and intentional traumatic injuries among urban Black men: A qualitative study. Injury.

[9] Li M., Wang Y., Chen H., Zheng F., Su Z., Li J., Yan H. (2025). Research on the anxiety and depression of patients with mechanical ocular injuries: A cross-sectional study. Psychol. Res. Behav. Manag..

[10] Karaman S., Ozkan B., Gok M., Karakaya I., Kara O., Altintas O., Altintas L. (2017). Effect of eye trauma on mental health and quality of life in children and adolescents. Int. Ophthalmol..

[11] Lu S., Li H., Yang X., Ma C., Li X. (2025). Epidemiology of ocular trauma and predictive modeling of visual outcomes: A 12-year retrospective study at a tertiary hospital in China. Risk Manag. Healthc. Policy.

[12] Elliott D.B. (2016). The good (logMAR), the bad (Snellen) and the ugly (BCVA, number of letters read) of visual acuity measurement. Ophthalmic Physiol. Opt..

[13] Caltrider D., Gupta A., Tripathy K. (2025). Evaluation of Visual Acuity. StatPearls.

[14] Shah M., Sundar G., Shah S. (2019). Ocular Trauma Score revisited—Making sense of it all. Lat. Am. J. Ophthalmol..

[15] Beck A.T., Ward C., Mendelson M., Mock J., Erbaugh J. (1961). Beck Depression Inventory (BDI). Arch. Gen. Psychiatry.

[16] Hisli N. (1988). Beck Depresyon Envanteri’nin geçerliliği üzerine bir çalışma. Psikoloji Dergisi.

[17] Beck A.T., Epstein N., Brown G., Steer R. (1988). An inventory for measuring clinical anxiety: Psychometric properties. J. Consult. Clin. Psychol..

[18] Ulusoy M., Sahin N.H., Erkmen H. (1998). Turkish version of the Beck Anxiety Inventory: Psychometric properties. J. Cogn. Psychother..

[19] Weathers F.W., Litz B.T., Herman D.S., Huska J.A., Keane T.M. The PTSD Checklist (PCL): Reliability, validity, and diagnostic utility. Proceedings of the Annual Convention of the International Society for Traumatic Stress Studies.

[20] Çorapçıoğlu A., Yargıç İ., Geyran P., Kocabaşoğlu N. (2006). Validity and reliability of the Turkish version of the Impact of Event Scale-Revised (IES-R). Nöropsikiyatri Araştırmaları.

[21] Ucan Gunduz G., Yalcinbayir O., Gullulu Z.Z., Ozkaya G. (2021). Clinical outcomes of posterior segment intraocular foreign bodies: The volume effect. J. Fr. D’ophtalmologie.

[22] Haagsma J.A., Ringburg A.N., van Lieshout E.M., van Beeck E.F., Patka P., Schipper I.B., Polinder S. (2012). Prevalence, predictors and long-term course of probable posttraumatic stress disorder after major trauma: A prospective cohort study. BMC Psychiatry.

[23] Baker R.S., Wilson M.R., Flowers C.W., Lee D.A., Wheeler N.C. (1996). Demographic factors in a population-based survey of hospitalized work-related ocular injury. Am. J. Ophthalmol..

[24] Barker-Collo S., Theadom A., Ameratunga S., Jones K., Jones A., Starkey N., Feigin V.L. (2013). Prevalence and predictors of post-traumatic stress disorder in adults one year following traumatic brain injury: A population-based study. Brain Impair..

[25] Liu Y., Peng S., Wu X., Liu Z., Lian Z., Fan H., Kuang N., Gu X., Yang S., Hu Y. (2025). Neural, cognitive and psychopathological signatures of a prosocial or delinquent peer environment during early adolescence. Dev. Cogn. Neurosci..

[26] Chotprasert N., Shrestha B., Thanasapburachot P., Kanpiputana R., Sipiyaruk K. (2022). Psychosocial distress and psychological adjustment in patients with ocular loss: A framework analysis. BMC Oral. Health.

[27] Fan H., Wang H., Lian Z., Yu Q., Wu X., Kuang N., Becker B., Feng J., Fan M., Song L. (2026). Dynamic Interactions Between Hemispheres Reveal a Compensatory Pathway for Motor Recovery in Moderate-to-Severe Subcortical Stroke. J. Stroke.

